# Supplementary material for: Band-selective Holstein polaron in Luttinger liquid material A0.3MoO3 (A = K, Rb)
Source: Nat Commun. 2021 Oct 26;12:6183. doi: 10.1038/s41467-021-26078-1 (PMC8548323; doi:10.1038/s41467-021-26078-1)
Supplement: Supplementary file 1 — Supplementary Information [file 41467_2021_26078_MOESM1_ESM.pdf]

Supplementary Information for

"Band-selective Holstein Polaron in Luttinger Liquid Material $A_{0.3}MoO_3$ (A = K, Rb)"

L. Kang[1*], X. Du[1*], J. S. Zhou[1], X. Gu[1], Y. J. Chen[1], R. Z. Xu[1], Q. Q. Zhang[1], S. C. Sun[1], Z. X. Yin[1], Y. W. Li[2,3], D. Pei[4], J. Zhang[2], R. K. Gu[4], Z. G. Wang[5], Z. K. Liu[2,3], R. Xiong[6], J. Shi[6], Y. Zhang[5], Y. L. Chen[1,2,3,4‡], and L. X. Yang[1,7‡]

[1]*State Key Laboratory of Low Dimensional Quantum Physics, Department of Physics, Tsinghua University, Beijing 100084, China.*

[2]*School of Physical Science and Technology, ShanghaiTech University and CAS-Shanghai Science Research Center, Shanghai 201210, China.*

[3]*ShanghaiTech Laboratory for Topological Physics, Shanghai 200031, China.*

[4]*Department of Physics, Clarendon Laboratory, University of Oxford, Parks Road, Oxford OX1 3PU, UK.*

[5]*International Center for Quantum Materials, School of Physics, Peking University, Beijing 100871, China.*

[6]*Department of Physics, Wuhan University, Wuhan 430072, China.*

[7]*Frontier Science Center for Quantum Information, Beijing 100084, China.*

[*]*These authors contribute equally to this work.*

[‡]*Email address: LXY: lxyang@tsinghua.edu.cn, YLC: yulin.chen@physics.ox.ac.uk*

This file includes the following contents:

Supplementary Figure 1-8.

Supplementary Note 1: Resistivity measurements on $A_{0.3}MoO_3$ (A = K, Rb);

Supplementary Note 2: X-ray diffraction measurements on $Rb_{0.3}MoO_3$;

Supplementary Note 3: *Ab initio* calculation of band structure;

Supplementary Note 4: Comparison between the calculated and measured band structure;

Supplementary Note 5: Fits to the data collected with He lamp;

Supplementary Note 6: Fits to the data below T_{CDW};

Supplementary Note 7: Evolution of band dispersion with surface Rb doping;

Supplementary Note 8: Estimation of the electron-phonon coupling parameter λ.

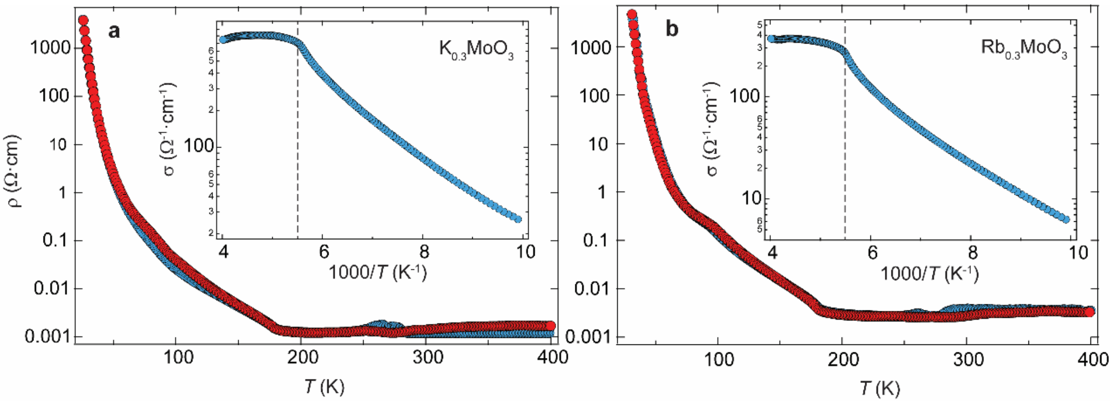

Supplementary Figure 1. **Temperature-dependent electrical resistivity measurements.** Data shown are measured on (**a**) $K_{0.3}MoO_3$ and (**b**) $Rb_{0.3}MoO_3$. The red and blue circles are the data collected during warming-up and cooling-down, respectively. The insets show the log-plot of the conductivity as a function of 1000/*T*. The CDW transition is observed near 183 K (vertical dashed lines) in both materials.

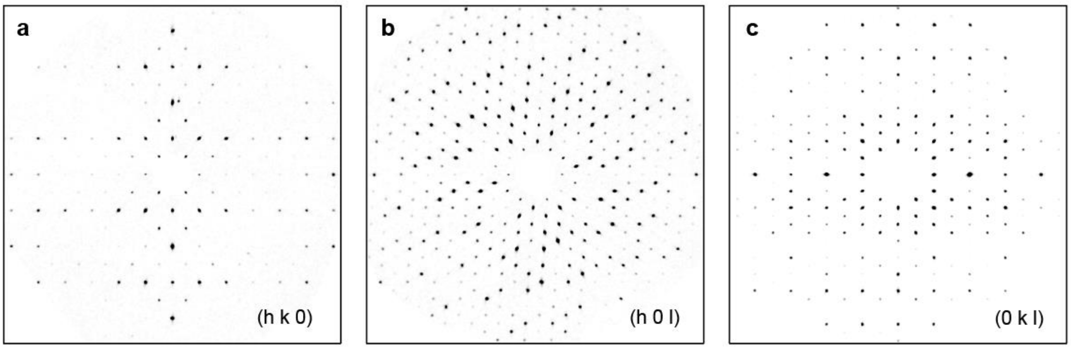

Supplementary Figure 2. **X-ray diffraction measurements.** The results shown are pattern of $Rb_{0.3}MoO_3$ along (**a**) (h k 0), (**b**) (h 0 l), and (**c**) (0 k l) planes.

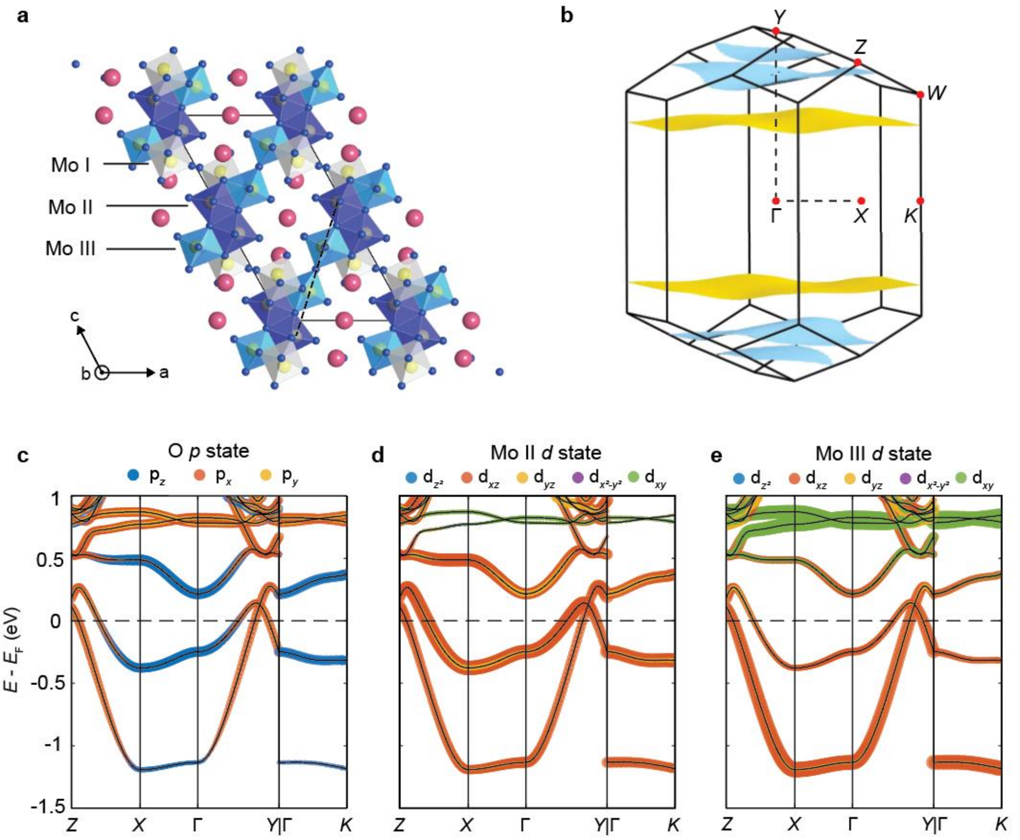

Supplementary Figure 3. **Calculated band structure of $K_{0.3}MoO_3$. a** The crystal structure of $A_{0.3}MoO_3$ with three inequivalent Mo atoms indicated. The black box indicates a unit cell and the dashed line marks the x axis adopted in atomic-orbital projected band structure calculation. **b** Calculated Fermi surface in the three-dimensional first Brillouin zone. **c-e** The band structure is projected onto (**c**) O *p* orbitals, (**d**) *d* orbitals of Mo II atoms, and (**e**) *d* orbitals of Mo III atoms. The dashed lines indicate the Fermi level (E_F).

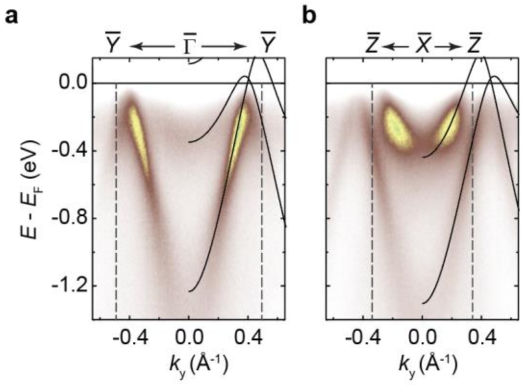

Supplementary Figure 4. **Comparison of the calculated (black curves) and measured band dispersions.** The comparison are plotted along (**a**) $\bar{\Gamma}\bar{Y}$ and (**b**) $\bar{X}\bar{Z}$. The dashed lines indicate the high symmetry points of the surface Brillouin zone.

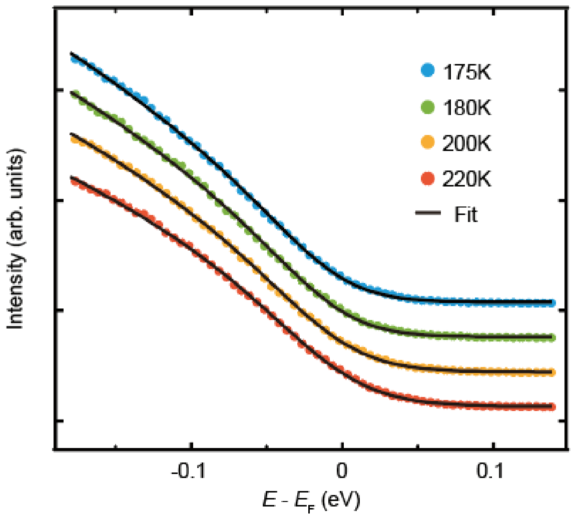

Supplementary Figure 5. **Fits to the data collected with He lamp using LL model at different temperatures.**

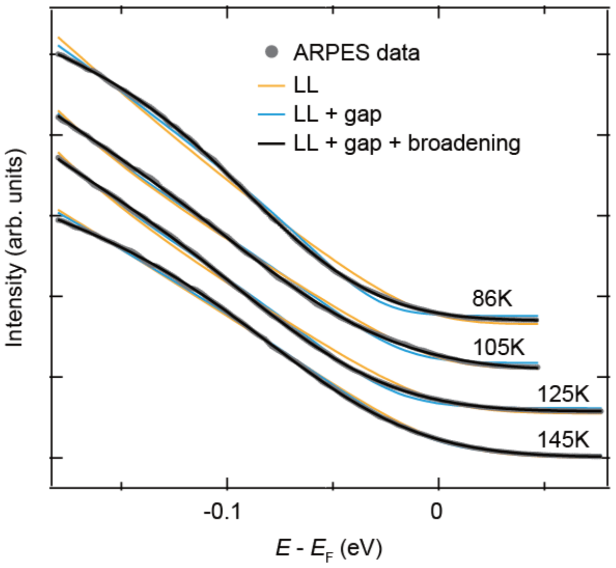

Supplementary Figure 6. **Data fits to the modified LL spectral function at temperatures below T_{CDW}.**

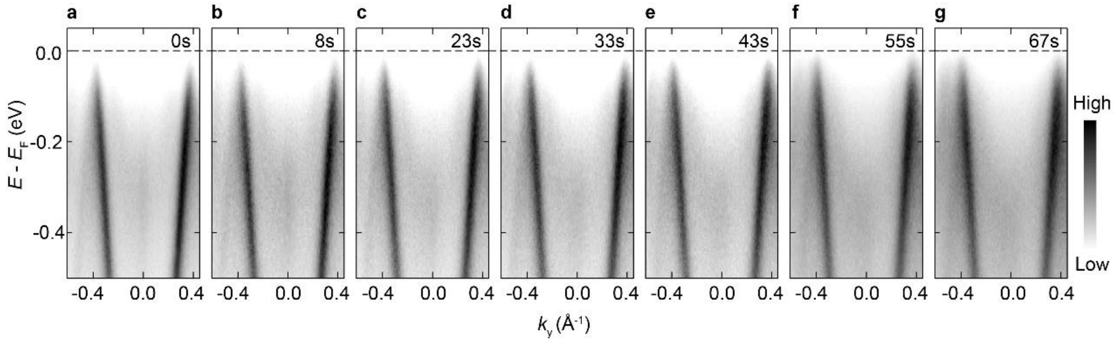

Supplementary Figure 7. **Evolution of band structure of $K_{0.3}MoO_3$ with surface Rb doping.** **a** Band structure of pristine $K_{0.3}MoO_3$. **b-g** Evolution of the band structure with subsequent Rb doping. Data were collected at 81 K using He lamp ($h\nu = 21.2$ eV). The dashed lines indicate E_F.

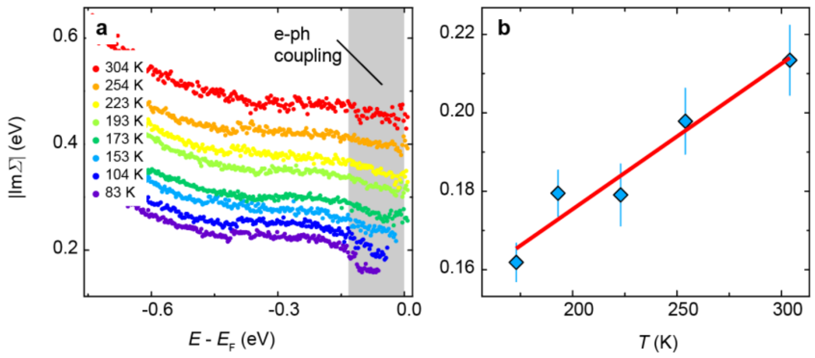

Supplementary Figure 8. **Strong electron-phonon coupling in $K_{0.3}MoO_3$. a** ImΣ derived from MDC width of the *B* band at different temperatures. Each curve is sequentially offset for 30 meV (from 83 K data) for clarity. **b** Temperature-dependence of ImΣ near E_F (blue diamonds). Red line is the linear fit to the data. The error bars are determined by the combination of confidence interval of data fitting and deviations between samples.

Supplementary Note 1: Resistivity measurements on $A_{0.3}MoO_3$ (A = K and Rb)

Supplementary Figures 1**a** and **b** show the resistivity as a function of temperature measured on single crystals of $K_{0.3}MoO_3$ and $Rb_{0.3}MoO_3$. The resistivity data are in good consistency with previous reports. The insets show the log-plots of the conductivity as a function of temperature, in which we can observe the CDW transition near 183 K in both samples. From the fits to the data with a standard model of the conductivity of a semiconductor, $\sigma \propto \exp(-\frac{\Delta}{2k_BT})$, we obtain a CDW gap of $2\Delta_{CDW}$ = 85 ± 15 meV, which is in good agreement with previous results and our ARPES measurement in the main text.

Supplementary Note 2: X-ray diffraction measurements on $Rb_{0.3}MoO_3$

The crystals used in our ARPES measurements have been characterized with X-ray diffraction (XRD) measurements. Supplementary Figure 2 shows sharp XRD patterns along different planes on $Rb_{0.3}MoO_3$, suggesting the high quality of our samples. From the XRD data, we obtain the lattice constants of a = 18.6635(17) Å, b = 7.5735(4) Å, c = 10.1075(9) Å, α = 90.0º, β = 118.767 º, and γ = 90.0 º, in good agreement with previous measurements (ICSD 73510) [1].

Supplementary Note 3: *Ab initio* calculation of the band structure

In Supplementary Figure 3, we present *ab initio* calculated band structure of $K_{0.3}MoO_3$. In Supplementary Figure 3b, the calculated Fermi surface is shown in the first Brillouin zone, which clearly demonstrates the Q1D characteristic of the electronic structure. Supplementary Figures 3c-e show band dispersions along high-symmetry lines together with the contributions of O and Mo atomic orbitals. The projected band dispersions suggest that the low energy states are almost exclusively composed of Mo d orbitals (mostly the Mo atoms in the center of the chains, i.e. Mo II and Mo III as indicated in Supplementary Figure 3a) and O p orbitals. More precisely, the bonding (B) and anti-bonding (AB) bands both exhibit strong Mo d_{xz} and O p_x character, while the AB band gains more O p_z character near the band bottom (the coordinate

system is chosen so that z axis is along the chain direction, and x axis is along the black dashed line in Supplementary Figure 3a).

Supplementary Note 4: Comparison between the calculated and measured band structure;

Supplementary Figure 4 compares the measured band structure with *ab initio* calculated results. After the calculated band structure is shift downward for about 100 meV, the calculation nicely reproduces the experimental band structure as shown by the overlaid black curves.

Supplementary Note 5: Fits to the data collected with He lamp

In addition to the data collected by 7eV laser (Fig. 3b in the main text), we also fit the data collected with He lamp ($h\nu$ = 21.2 eV) to the LL model as shown in Supplementary Figure 5. The EDCs are integrated in a momentum range of [-0.65, 0.65] Å^{-1}, much larger than that in data collected with 7 eV laser. The data again fit perfectly to the LL model with $\alpha = 0.6 \pm 0.1$, confirming the LL phase of the normal state of blue bronzes.

Supplementary Note 6: Fits to the data below T_{CDW}

At temperatures slightly below T_{CDW}, the EDCs can still be well fitted by the LL model, suggesting the intimate relationship between the CDW state and the LL phase in the normal state. As the temperature further decreases, the data cannot be fitted by the LL model anymore and a phenomenological gap Δ has to be included in the spectral function:

$$\rho(\omega, T) \propto T^{\alpha}\mathrm{Re}\left[(2\mathrm{i})^{\alpha+1}\mathrm{B}\left(\frac{\alpha + 1 + \frac{\mathrm{i}(\omega - \Delta)}{\pi k_B T}}{2}, -\alpha\right)\right], \quad (1)$$

As shown by the blue curves in Supplementary Figure 6, the data at 145 K and 125 K can be better fitted using the modified LL model. However, far below T_{CDW}, at 105 K and 86 K, the data cannot be fitted by either the LL or the modified LL model, suggesting the strong modification of the spectra by the CDW ordering.

Supplementary Note 7: Evolution of band dispersion with surface Rb doping.

Supplementary Figure 7 shows the evolution of the band structure of $K_{0.3}MoO_3$ with surface doping of Rb atoms at 80 K. We observe a clear upward shift of the band edge, suggesting the suppression of the CDW gap by surface doping. After the third doping sequence, the EDC leading edge shifts for about 20 meV and saturates in the subsequent doping processes [Figs. 3e, f in the main text].

Supplementary Note 8: Estimation of the electron-phonon coupling parameter λ.

To estimate the electron-phonon coupling (EPC) constant λ, we further investigate electron self-energy [$\mathrm{Im}\Sigma(E) = v_0 \cdot \Delta k(E)/2$, where v_0 is the bare band velocity and $\Delta k(E)$ is the linewidth of the MDCs] of the B band at different temperatures as shown in Supplementary Figure 8a[2]. Despite the LL nature of blue bronzes, we can roughly estimate the EPC constant using the approximation that $\mathrm{Im}\Sigma(E)$ near E_F depends linearly on the temperature at high enough temperatures[3, 4, 5, 6], $\mathrm{Im}\Sigma(0,T) = \lambda\pi k_B T$, considering that the electron-phonon self-energy of a LL described by Holstein model is similar to that of a Fermi liquid[7]. Supplementary Figure 8b presents $\mathrm{Im}\Sigma(E)$ integrated in an energy window of 30 meV near E_F as a function of temperature, showing a linear dependence with temperature. The total EPC constant λ_{tot} is estimated to be 1.2 ± 0.2, consistent with previous theoretical and experimental results[8, 9, 10, 11, 12]. Taking the contribution of lattice thermal fluctuation induced by phase phonons into account, the dimensionless EPC parameter λ is about 0.54 ± 0.2, in good agreement with our simulation in the main text.

References:

1. Schutte WJ, de Boer JL. The incommensurately modulated structures of the blue bronzes K0.3MoO3 and Rb0.3MoO3. *Acta Crystallogr B Struct Sci Cryst Eng Mater* **49**, 579-591 (1993).

2. Mou D, Konik RM, Tsvelik AM, Zaliznyak I, Zhou X. Charge-density wave and one-dimensional electronic spectra in blue bronze: Incoherent solitons and spin-charge separation. *Phys Rev B* **89**, 201116 (2014).

3. Matetskiy AV*, et al.* Two-Dimensional Superconductor with a Giant Rashba Effect: One-Atom-Layer Tl-Pb Compound on Si(111). *Phys Rev Lett* **115**, 147003 (2015).

4. Pan ZH, Fedorov AV, Gardner D, Lee YS, Chu S, Valla T. Measurement of an exceptionally weak electron-phonon coupling on the surface of the topological insulator Bi_2Se_3 using angle-resolved photoemission spectroscopy. *Phys Rev Lett* **108**, 187001 (2012).

5. Shen L*, et al.* Evolution of electronic structure and electron-phonon coupling in ultrathin tetragonal CoSe films. *Phys Rev Mater* **2**, 114005 (2018).

6. Valla T, Fedorov AV, Johnson PD, Hulbert SL. Many-Body Effects in Angle-Resolved Photoemission: Quasiparticle Energy and Lifetime of a Mo(110) Surface State. *Phys Rev Lett* **83**, 2085-2088 (1999).

7. Assaad FF. Spin, charge, and single-particle spectral functions of the one-dimensional quarter filled Holstein model. *Phys Rev B* **78**, 155124 (2008).

8. Degiorgi L*, et al.* Fluctuation effects in quasi-one-dimensional conductors: Optical probing of thermal lattice fluctuations. *Phys Rev B* **52**, 5603-5610 (1995).

9. Mankowsky R*, et al.* Dynamical Stability Limit for the Charge Density Wave in K0.3MoO3. *Phys Rev Lett* **118**, 116402 (2017).

10. Beyer R, Barišić N, Dressel M. Charge-density fluctuations probed by vibronic modes of $K_{0.3}MoO_3$. *Physica B Condens Matter* **407**, 1823-1826 (2012).

11. Yang LX*, et al.* Bypassing the Structural Bottleneck in the Ultrafast Melting of Electronic Order. *Phys Rev Lett* **125**, 266402 (2020).

12. Degiorgi L, Gruner G, Kim K, McKenzie RH, Wachter P. Optical probing of thermal lattice fluctuations in charge-density-wave condensates. *Phys Rev B Condens Matter* **49**, 14754-14757 (1994).
